# Host and bacterial factors linking periodontitis and rheumatoid arthritis

**DOI:** 10.3389/fimmu.2022.980805

**Published:** 2022-08-25

**Authors:** Anna Krutyhołowa, Karolina Strzelec, Agata Dziedzic, Grzegorz P. Bereta, Katarzyna Łazarz-Bartyzel, Jan Potempa, Katarzyna Gawron

**Affiliations:** ^1^ Department of Microbiology, Faculty of Biochemistry, Biophysics and Biotechnology, Jagiellonian University, Krakow, Poland; ^2^ Department of Molecular Biology and Genetics, Faculty of Medical Sciences in Katowice, Medical University of Silesia, Katowice, Poland; ^3^ Department of Periodontology and Oral Medicine, Faculty of Medicine, Medical College, Jagiellonian University, Krakow, Poland; ^4^ Department of Oral Immunology and Infectious Diseases, School of Dentistry, University of Louisville, Louisville, KY, United States

**Keywords:** periodontitis, rheumatoid arthritis, oral microbiome, citrullination, ACPA, autoimmune disease, infection

## Abstract

Observations from numerous clinical, epidemiological and serological studies link periodontitis with severity and progression of rheumatoid arthritis. The strong association is observed despite totally different aetiology of these two diseases, periodontitis being driven by dysbiotic microbial flora on the tooth surface below the gum line, while rheumatoid arthritis being the autoimmune disease powered by anti-citrullinated protein antibodies (ACPAs). Here we discuss genetic and environmental risk factors underlying development of both diseases with special emphasis on bacteria implicated in pathogenicity of periodontitis. Individual periodontal pathogens and their virulence factors are argued as potentially contributing to putative causative link between periodontal infection and initiation of a chain of events leading to breakdown of immunotolerance and development of ACPAs. In this respect peptidylarginine deiminase, an enzyme unique among prokaryotes for *Porphyromonas gingivalis*, is elaborated as a potential mechanistic link between this major periodontal pathogen and initiation of rheumatoid arthritis development.

## Introduction

Periodontal diseases affect the gingiva, the supporting connective tissue and the alveolar bone. They are one of the most common inflammatory disorders, affecting nearly 30% of the population worldwide (1). Specifically, two diseases can be distinguished: the first is gingivitis, which is inflammation of the gingiva and is limited to the soft-tissue compartment of the gingival epithelium and connective tissue; the second is periodontitis (PD), defined as inflammation of the tooth-supportive tissues, which results in attachment loss and bone destruction. Many years of research have identified a number of microbial aetiologies for periodontal diseases. Socransky and Haffajee (2) summarized the hypotheses regarding possible causes and postulated that periodontal diseases are triggered by infection. The oral cavity is colonized by many different microorganisms. More than 700 species of oral bacteria have been identified in biofilms (dental plaque) (3). Extensive analysis of dental plaques has resulted in a detailed description of polymicrobial communities associated with general health or periodontal disease (2, 4–10). These studies also re**-**classified PD as a microbial shift disease in which a Gram-positive microbiota shifts to a mostly Gram-negative microbiota (11). However, the exact mechanisms underlying changes in microbial composition remain unclear. Recent metagenomic and mechanistic studies suggest that PD is not caused by the presence of a few specific periodontal pathogens; rather, it results from polymicrobial synergy and dysbiosis (12–16). Thus, the concept of polymicrobial synergy and dysbiosis was proposed (17). Many studies report an association between PD and three periodontal pathogens, namely, *Porphyromonas gingivalis (P. gingivalis)*, *Tannerella forsythia (T. forsythia)* and *Treponema denticola (T. denticola)* (designated as “red complex” bacteria). Recent whole genome DNA probe studies of the oral microbiota identified new bacterial species that correlate with PD, namely, *Aggregatibacter actinomycetemcomitans (A. actinomycetemcomitans)*, *Filifactor alocis* (*F. alocis*) and other species belonging to the genera *Peptostreptococcaceae*, *Desulfobulbus* and *Synergistetes* (12, 18–20). The presence of oral polymicrobial communities cannot stay unnoticed by the immune system. Innate defence mechanisms regulate the composition of the microbiome and help to maintain periodontal health. For example, healthy periodontal tissue is characterized by a high number of neutrophils transiting through the junctional epithelium and expression of numerous host innate mediators, such as defensins (BD1, BD2 and BD3), cytokines (IL-8) and lipopolysaccharide-binding protein (LBP) (21–23). Moreover, even though cytokines associated with tissue damage, *i.e.*, interleukin 1β (IL-1β), tumour necrosis factor α (TNFα) and prostaglandin E_2_ (PGE_2_) are present in gingival crevicular fluid (GCF) from clinically healthy sites, these levels are lower than those in fluid from diseased sites (24, 25). Innate immune mechanisms make a marked contribution to bone resorption in PD. This process is controlled by the ratio of RANKL (receptor activator of nuclear factor-κB ligand), which induces differentiation of osteoclast precursors, to osteoprotegerin (OPG), which is a soluble receptor for RANKL (26, 27). RANKL expression is regulated by proinflammatory cytokines, such as TNFα and IL-1β, thus an increase in the concentration of these cytokines in healthy tissue can lead to bone loss (28, 29). Both, commensal and pathogenic bacteria activate innate immune responses *via* Toll-like receptors, which are responsible for recognition of bacterial components. This suggests that a microbiome of appropriate composition ensures precise equilibrium between native immune responses within healthy tissues. Dysbiosis or a microbiome shift may disturb the balance between expression of inflammatory and anti-inflammatory mediators and lead to destruction of bone and tooth-supporting tissues. Hence, PD results from the combined influence of a dysbiotic microbiome that forms dental plaques and host inflammatory responses that destroy the periodontium. Many studies demonstrate a central role for *P. gingivalis* in pathogenesis of PD, particularly since this bacterium can cause bone loss after implantation into the oral cavity of animals (30–32). However, recent studies show that the pathogen itself does not cause PD in mice lacking commensal bacteria (15). This suggests, that the actual role of *P. gingivalis* is to manipulate host responses and convert a symbiotic community into a dysbiotic one, resulting in destructive inflammation. It should be noted, however, that species, such as *T. forsythia*, *T. denticola* and *A. actinomycetemcomitans* can modulate host immune responses, suggesting that they also impact on PD (33–35).

**Figure 1:**
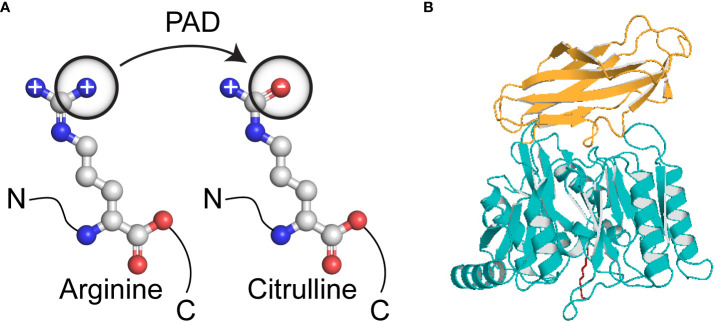
Conversion of arginine to citrulline. **(A)** Biochemical reaction resulting in loss of positive charge on a side chain; **(B)** The crystallised structure of *Porphyromonas gingivalis* peptidylarginine deiminase comprises two domains; deiminase domain aa490-360 (yellow) and Ig-like fold aa361-461 (blue). The substrate in the active site was marked as red.

Rheumatoid arthritis (RA) is a systemic autoimmune disease that affects 0.5–1% of the population worldwide (36). It is characterized by chronic inflammation of synovial joints and bone erosion, which together result in joint destruction, disability, susceptibility to other pathological conditions and shorter life expectancy. The aetiology of the disease remains unknown, but many studies suggest involvement of both, genetic and environmental factors; indeed, genetic influences contribute to approximately 60% of RA cases (37). Importantly, an infectious agent in a susceptible host may be a trigger factor for this disease (38). Rheumatoid factor (RF), an antibody specific for the Fc fragment of IgG, is an established diagnostic marker for RA. Thus, IgG antibodies were considered major autoantigens in RA. However, further studies identified RF in other autoimmune and infectious diseases, as well as in 5% of the healthy population; in these cases, it may be a response to polyclonal B cell activation (39). More recent studies focused on the role of citrullinated proteins and peptides as possible autoantigens in RA. Citrullination, also known as deimination, is a post**-**translational enzymatic modification that converts a positively charged arginine residue into a neutral citrulline residue (**Figure 1**). This reaction is catalyzed by calcium ion**-**dependent enzymes called peptidylarginine deiminases (PADs) (40). The human genome encodes five members of the PAD family (PAD1, PAD2, PAD3, PAD4 and PAD6), which differ with respect to tissue distribution, cellular sub**-**localisation and substrate specificity (41). Many studies confirmed that citrullination is required for physiological processes, such as citrullination of keratin and filaggrin during keratinocyte differentiation or for citrullination of myelin basic protein, which ensures electrical insulation of myelin sheets (42–44). There are also strong suggestions that citrullination plays a role in transcriptional regulation and chromatin decondensation during neutrophil trap formation (45). However, extensive or inadequate deimination can lead to pathological conditions. Replacement of arginine, which often plays a central role in the structural and functional integrity of proteins, may alter protein folding, thereby exposing new epitopes to the immune system. PAD2 and PAD4 seem to play a crucial role in RA due to their presence in the rheumatoid synovial membrane, synovial fluid cells and synovial fluid (46–49). To date, four well established citrullinated autoantigens have been identified, *i.e.*, collagen type II, fibrinogen, vimentin and α-enolase (50–53). All of these proteins can be found in the joints and all can form immune complexes with antibodies, which then mediate further inflammatory reactions.

## Epidemiologic correlation between PD and RA

A scientific concept claiming that dental sepsis can cause systemic inflammation, including arthritis, arose in the 19^th^ century and was developed further in the 20^th^ century. Despite the fact, that in 1952 the American Medical Association pronounced that tooth extraction was not based on scientific evidence, and that the practice should not be considered as a treatment approach to reduce the severity or symptoms of RA, the method was used continuously up until the 1970s, when the first effective RA drugs, *e.g.*, penicillamine appeared (54–56).

Recently, links and associations between PD and RA have been investigated intensively and many studies show a correlation between these two diseases (57–60). The earliest studies investigated the prevalence of RA in patients with PD. Mercado et al. (61) showed that individuals with moderate to severe PD are more susceptible to RA. They examined 1412 individuals attending a dental clinic and divided them into two groups, *i.e.*, those with PD and a control group comprising individuals attending the dental clinic for general treatment. The prevalence of RA in the PD group was significantly higher than that in the control group. Moreover, patients with RA were more likely to have a moderate to severe form of PD than patients without RA. However, it should be kept in mind that this study relied on self-reported RA and used non**-**validated parameters to classify PD.

Subsequent studies tried to dissect whether PD occurs more often in patients with established RA. Most studies used criteria for PD defined by the American Association of Periodontology and criteria for RA defined by the American College of Rheumatology (62). To evaluate the association between PD and RA, several factors were analysed, namely, genetic factors, proinflammatory factors and the presence of different oral bacterial DNA species in periodontal pocket samples, serum and synovial fluids from patients with RA (63). PD severity was identified as the third most potent predictor of RA, immediately after female gender and smoking (57). Occurrence of PD correlated strongly with RA, hence it was proposed that both diseases are driven by a common molecular mechanism.

PD and RA are characterized by chronic inflammation, and TNFα is considered a major proinflammatory mediator. The main source of TNFα in RA are joint macrophages (64). A study by Nilsson et al. (65) shows that plasma levels of TNFα are related to the degree of systemic inflammation and may affect PD development in patients with RA. Patients suffering from RA that have higher levels of circulating TNFα show worse clinical parameters with respect to the periodontium, *i.e.*, enhanced bleeding on probing, lower clinical attachment level and greater probing pocket depth. However, the association between TNFα and PD is not exclusive to RA. Increased levels of TNFα are also associated with severity of PD in patients with diabetes type 2 (66). One problem with studies evaluating levels of proinflammatory mediators in PD and RA is treatment of RA symptoms during the study. In such cases, lack of higher levels of proinflammatory mediators, such as TNFα and/or C-reactive protein in patients suffering from both PD and RA is likely due to pharmacologic treatment of RA (65).

## “Risk factors” that link PD and RA

**Figure 2:**
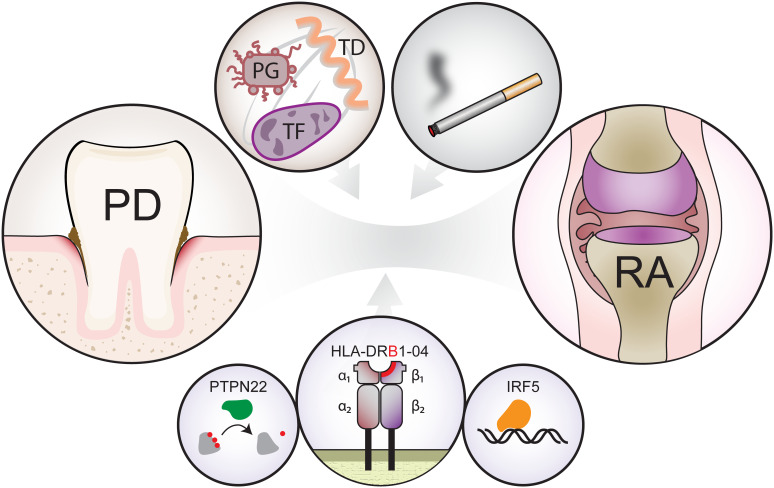
Shared risk factors that contribute to development of periodontal disease (left circle, PD) and rheumatoid arthritis (right circle, RA). The top left circle shows bacteria forming the “red complex”, namely, *Porphyromonas gingivalis* (red, PG), *Tannerella forsythia* (violet, TF) and *Treponema denticola* (orange, TD) and the top right circle shows an environmental factor (smoking). Bottom three circles show the main genetic risk factors, namely shared epitopes within the β-chain of human leukocyte antigen (HLA), tyrosine phosphatase (PTPN22) and Interferon Regulatory Factor 5 (IRF5).

PD and RA are similar in several ways. Both diseases are characterized by chronic inflammation, which leads to tissue destruction. The aetiology of both diseases is multifactorial and includes genetic and environmental components (**Figure 2**). Although the hypothesis that bacteria are the main cause of PD is well established, there is no direct proof that RA has a microbial origin. By contrast, an evidence exists that autoimmunity plays a role in PD (67). Some studies report detection of antibodies specific for host components, such as collagen and DNA, as well as aggregation of antibodies and increased lymphotoxicity toward oral epithelial cells and fibroblasts (67).

The main genetic risk factor for autoimmune diseases is related to major histocompatibility complex class II (MHC II) molecules, certain alleles of which may strongly favour presentation of citrullinated epitopes and promote generation of autoimmune antibodies. The most significant alleles responsible for RA susceptibility are present on HLA**-**DR4, **-**DR1 and **-**DR10. These genetic variants mainly affect six amino acids, namely, EQKRAA, situated in the peptide-binding cleft of the MHC II molecule. These alleles, collectively called “shared epitopes”, are important for RA development (68, 69). Shared epitopes are associated mainly with the presence of antibodies against citrullinated proteins rather than with the disease itself (70). In particular, HLA-DR4 is associated with rapidly progressive PD; in patients with PD, the frequency of shared epitopes is higher than that in controls (71, 72).

In addition, genetic variants of tyrosine phosphatase (PTPN22), which may be involved in regulating T cell and B cell activation may contribute to a connection between PD and RA (73, 74). Another study suggests, that PD and RA share interferon regulatory factor 5 (IRF5) and PR domain zinc finger protein 1 (PRDM1) as susceptibility factors (75). Also, epigenetic alterations within the X chromosome may, at least partially, explain the higher prevalence of RA in women with PD (76).

Smoking is considered an environmental risk factor for both PD and RA. Epidemiological studies of large cohorts show significant association between smoking and RA (77). Indeed, smoking stimulates peptide citrullination by PAD2 and PAD4 (78). This finding strongly supports the concept of pathological importance of anti**-**citrullinated protein antibodies (ACPAs) in development of ACPA**-**positive RA, however, smoking**-**induced citrullination fails to explain ACPA**-**negative RA. Smoking worsens both PD and RA by promoting growth of bacteria. A study of patients with PD where smokers were distinguished from non**-**smokers showed, that smoking was associated with increased load of *T. forsythia*, *Peptostreptococcus micros (P. micros)*, *Fusobacterium nucleatum (F. nucleatum)* and *Campylobacter rectus (C. rectus)* in subgingival dental plaque (79).

Another factor that may affect bacterial growth is infection with Epstein**-**Barr virus (EBV**-**1) and cytomegalovirus. These viruses promote colonization of the gingiva by *P. gingivalis*, *A. actinomycetemcomitans*, *T. forsythia*, *Prevotella intermedia (P. intermedia)*, *Prevotella nigrescens (P. nigrescens)* or *T. denticola* (80). PCR**-**based studies of gingival tissue detected EBV in 71–89% and cytomegalovirus in 65–78% of patients with severe PD, but in only 6% of healthy control subjects (81).

## Impact of periodontal pathogens on pathogenesis of PD and RA

Periodontal bacteria play a crucial role in progression of PD and may be a factor linking this disease with RA. This thesis is supported by several studies showing, that serum and synovial fluid from RA patients contain higher levels of antibodies against periodontal pathogens than serum from control subjects. The detected antibodies were specific for *P. gingivalis*, *P. intermedia*, *Prevotella melaninogenica (P. melaninogenica)* and *T. forsythia* (82, 83). In addition to antibodies, DNA from periodontal bacteria was detected in serum and synovial fluids from RA patients. Martinez**-**Martinez et al. (84) detected periodontal bacterial DNA in 100% of synovial fluid samples and in 83.5% of serum samples from RA patients. The most common bacterial DNAs in synovial fluid were derived from *P. intermedia* (73.6%) and *P. gingivalis* (42.1%). However, synovial fluid samples from RA patients plated on agar did not show bacterial growth, and bacterial DNA was not detected in leukocytes. A similar result was reported by Moen et al. (85), who reported that the most common DNAs in synovial fluid and serum were derived from *P. intermedia*, *P. gingivalis* and *T. denticola*. Interestingly, there was no significant difference between the amount of bacterial DNA in subgingival dental plaques, serum and synovial fluid in cases with *A. actinomycetemcomitans* and *P. gingivalis.* By contrast, *P. intermedia*, *T. forsythia*, *P. nigrescens* and *T. denticola* were significantly more abundant in subgingival dental plaques than in serum and synovial fluid. It should be noted, that species detected in synovial fluid and serum were also present in subgingival dental plaques. Detection of bacterial DNA in synovial fluid suggests transport of DNA from periodontal tissue to the joints of RA patients. The mechanism underlying such transport is unclear, however, several hypotheses have been proposed: (i) transport of free DNA *via* the bloodstream, (ii) transport *via* viable non**-**immune cells and (iii) intracellular capture and transport by phagocytes and other immune cells (86). Many studies were conducted to elucidate the mechanism(s) underlying transport of bacterial DNA. These studies included testing lymphocytes isolated from blood for the presence of bacterial DNA and inoculation of synovial fluid onto different growth media under both, aerobic and anaerobic conditions, however, neither approach showed a positive result, suggesting, that bacterial DNA is transported in the blood as free DNA (84).

Another interesting study in patients with RA showed that oral and gut microbiota are different from those in healthy control subjects (87). The results revealed, that *P. gingivalis* was more common in control saliva and in control dental plaques, and that there was no association between this species, peptidylarginine deiminase levels and RA. These results are in agreement with those of other studies confirming a lack of correlation between *P. gingivalis* and RA (88, 89). By contrast, anaerobic species, such as *Lactobacillus salivarius*, *Atopobium* spp. and *Cryptobacterium curtum (C. curtum)* were enriched in both salivary and dental samples from subjects with RA, whereas aerobes, such as *Neisseria* spp. and *Rothia aeria* were enriched in control samples. Appropriate treatment of RA patients resulted in partial resolution of these alterations (87). A study conducted on periodontally healthy subjects with and without RA showed, that Gram**-**negative anaerobes were significantly more abundant in RA (90). Such a dysbiotic state could be an indication for further development of PD. *P. gingivalis* and *A. actinomycetemcomitans* were not dominant components of the microbiome, and there was no significant difference in their abundance between the groups. Therefore, it is likely, that other bacteria play a role in initiating RA, but *P. gingivalis* and *A. actinomycetemcomitans* contribute at later stages to maintain the disease. Interestingly, this study identified *C. curtum* in the periodontal microbiome of RA patients (100**-**fold greater abundance in RA, with 39**-**fold greater odds of detection) (90). The fact, that *C. curtum* is more abundant in the oral and gut microbiota of those with early RA does not imply an aetiopathogenic role for *C. curtum*, however, this species may be an interesting candidate for future studies. Moreover, PD is significantly more common in RA patients compared to healthy controls: the incidence is higher in people in the earliest stages of the disease, which is probably related to the role of *P. gingivalis* in inducing citrullination and the development of new antigens.

### P. gingivalis

*P. gingivalis* is an obligate, anaerobic, non**-**motile, asaccharolytic Gram**-**negative bacterium, that forms black**-**pigmented colonies on blood agar plates. It is found in 85.75% of subgingival plaque samples from patients with chronic PD (91). As an obligate anaerobe, *P. gingivalis* occupies dental pockets and is considered a secondary colonizer of dental plaques; secondary colonizers adhere to primary colonizers, such as *Streptococcus gordonii* and *P. intermedia*. There are invasive and non**-**invasive strains of *P. gingivalis*. This classification is based on their ability to form abscesses in a mouse model (91). *In vitro* and *in vivo* studies show that invasive strains are more pathogenic than non**-**invasive strains (92, 93). *P. gingivalis* harbours a wide variety of virulence factors, including fimbriae, capsules, lipopolysaccharide (LPS), lipoteichoic acids, haemagglutinins, gingipains, outer membrane proteins, outer membrane vesicles and peptidylarginine deiminase (PPAD) (94, 95). Expression of virulence factors is often regulated by changes in the external environment (96–98). Active virulence factors are responsible for manipulation of host immune responses, induction of host responses *via* cytokine production and inhibition of host protective mechanisms *via* citrullination and protease degradation, all of which lead to rapid destruction of periodontal tissues and bone resorption (95).

*P. gingivalis* has a unique ability to express a special PPAD, which is considered to be one of the virulence factors harboured by this periodontal pathogen (99). Biochemical studies show, that PPAD deiminates preferentially the C**-**terminal arginine of peptides and proteins, and in contrast to human PADs, it is able to citrullinate L**-**arginine in a calcium**-**independent manner (100). *P. gingivalis* strains lacking PPAD or harbouring an inactive form of this enzyme are less adherent and invade human gingival fibroblasts less efficiently than the wild**-**type strain (101). Moreover, mutant strains show an impaired ability to stimulate PGE_2_ **-**dependent signalling pathways, and these properties are restored by addition of purified enzyme to the cell cultures. Also, PPAD is an important factor that alters host immune responses. For example, citrullination of LL**-**37 and components of the complement system, *e.g.*, the C5a anaphylatoxin results in loss of function (102, 103). Moreover, in both, RA and PD, local inflammation of the gingival mucosa or the synovial membrane of the joint is exacerbated by the influx of inflammatory cells from the circulation. The conditions in the gingival pockets in combination with the biofilm, which includes *P. gingivalis*, increase the production of ACPA antibodies due to the high activity of PPAD. The above factors contribute to the stimulation of subsequent inflammatory processes.

PPAD is not the only virulence factor of *P. gingivalis* that has a possible role in initiating RA onset. Some of the most important virulence factors harboured by *P. gingivalis* are lysine/arginine-specific cysteine proteases, called gingipains. They play a major role in both, bacterial development and infection. They are responsible for maturation of fimbriae, which enable bacteria to attach to and invade host cells, such as human gingival fibroblasts and epithelial cells (104, 105). The major component of long fimbriae is the FimA protein. A recent study showed, that both, collagen-induced arthritis and PD in mice infected with *P. gingivalis* pre-incubated with an anti**-**FimA antibody were markedly less severe, suggesting that disrupting functional fimbriae with an anti**-**FimA antibody may ameliorate RA (106). As proteolytic enzymes, gingipains degrade host extracellular matrix proteins, such as laminin, fibronectin and collagen, as well as complement system components. Arginine**-**specific gingipain cooperates with PPAD, and the cleavage of the substrate leaves arginine as a C**-**terminal residue, which can then be modified by PPAD (107). Similar to *P. gingivalis*, other oral bacteria express extracellular proteases, which may reveal new antigens *via* proteolytic cleavage. The blood of individuals with pre**-**RA and established RA contains higher levels of anti**-**RgpB than that of healthy controls. Unlike ACPA, the level of which increases over time, the level of anti**-**RgpB decreases over time (108). In contrast, a study of a Southern European cohort revealed no association between pre-RA and anti-RgpB (109). However, the study did not evaluate periodontal status and anti**-** *P. gingivalis* antibody levels.

Moreover, some reports indicate that in RA patients DNA of *P. gingivalis* can be detected in the synovial fluid, and antibodies in serum against this bacterium. Additionally, the immunologic response against *P. gingivalis* is also present in people genetically predisposed to RA development in whom serum antibodies against citrullinated proteins and RF are detected. Importantly, there was no evidence of an immune response in these patients against other associated periopathogens with the infectious etiology of chronic periodontitis. *P. gingivalis*, as the only finely system, produces PPAD and the citrullination of proteins, which may be important in the development of an immune response against citrullinated proteins in RA patients, thus constituting a significant risk factor for the development of RA.

Most recently it was shown, that repeated oral infections with *P. gingivalis* directly caused development of seropositive arthritis accompanied by systemic inflammation and bone destruction in Lewis rats. Conversely, infection with *P. intermedia* resulted only in mild gingivitis but no bone erosion (110). The observed bone erosion strongly resembled pathological manifestation of RA in an adjuvant**-**induced arthritis model in rats (111). Moreover, the bone destruction pattern was consistent with results from human RA, which also showed an increase of IgM, anti**-**IgG and anti**-**CCP2 levels and their correlation with anti**-** *P. gingivalis* antibodies (83, 110).

### T. denticola

*T. denticola* is a common oral bacterium closely associated with both, PD and aetiology of implant**-**related periarthritis (112). This Gram**-**negative, obligatory anaerobic bacterium inhabits subgingival plaques. Numerous studies allowed identification of *T. denticola* virulence factors, which include leucine**-**rich repeat proteins, metabolic end**-**products, biofilm creation, toxin**-**antitoxin systems, dentilisin, lipoproteins, trypsin-like protease activity and sheath proteins (113–118). Unlike *P. gingivalis* and *T. forsythia*, *T. denticola* is motile and able to respond chemotactically to environmental changes (33). Several studies suggest, that the presence of *P. gingivalis* is required for colonization by *T. denticola*, however, interactions between “red complex” bacteria are unclear (119, 120). Mouse subcutaneous abscess models of disease have been used to investigate *T. denticola* virulence factors, as well as those of other species from that genus (121, 122). These studies showed that mono**-**infection with *Treponemes* may cause localised lesions, but only co**-**infection with *P. gingivalis* and *T. denticola* leads to increased tissue damage as compared with that after mono**-**infection by *P. gingivalis* (123). Although the relevance of these abscess models has been questioned, and more adequate models have been developed, more recent studies confirm these results. For instance, inoculation with the “red bacterial complex” at a 1:1:1 ratio resulted in greater bone resorption than mono-inoculations with the same total number of bacterial cells (124). At the same time, co**-**infection with *P*. *gingivalis* and *T. denticola* causes the same level of damage as a 40**-**fold higher number of *P. gingivalis* alone (125). These studies underline the importance of synergistic interactions of periodontal pathogens in development and severity of periodontal disease and probably with PD**-**associated diseases, such as RA.

### T. forsythia

*T. forsythia* is a Gram**-**negative, anaerobic bacterium and a member of the “red complex”. A growing body of evidence implicates *T. forsythia* in the pathogenesis of PD, however, the species is relatively understudied due to its demanding growth requirements and difficulties with genetic manipulations (126, 127). A number of studies demonstrated, that *T. forsythia* is the first colonizer of a dental plaque, and that it may be a necessary precursor species for *P. gingivalis* and *T. denticola* colonization. Moreover, *T. forsythia* is more likely to cause PD in overweight women than in women of normal weight (128). Recent studies show overgrowth of *T. forsythia* in overweight and obese individuals when compared with those of a normal weight (129). A study conducted on patients with RA showed that *T. forsythia* was associated with highly active RA and high salivary ammonium levels, which may be a result of PPAD activity (130). There are several putative virulence factors expressed by *T. forsythia*, *i.e.*, KLIKK proteases and PrtH protease, sialidases, a leucine**-**rich repeat cell surface**-**associated and secreted protein (BspA), a hemagglutinin, components of the bacterial S**-**layer and methylglyoxal production (131). To fully understand the *T. forsythia* virulence mechanisms that contribute to pathogenesis of PD and RA, future studies should be mostly focused on interaction between its virulence factors, other bacteria and host responses.

### A. actinomycetemcomitans

*A. actinomycetemcomitans* is a Gram**-**negative bacterium associated with aggressive PD, chronic PD and several non**-**oral infections (132). It is found in the oral cavity of more than one**-**third of the population (133, 134). This species harbours a wide variety of virulence factors, including adhesins, fimbriae, exotoxins and endotoxins, all of which vary among individual strains (serotypes). Certain serotypes are more prevalent in individuals suffering from aggressive PD (132). Its ability to produce leukotoxin**-**A (LtxA) is considered to be the most important virulence factor. This toxin kills white blood cells, which results in untamed bacterial growth and potent stimulation of the host immune response (135). LtxA from *A. actinomycetemcomitans* belongs to the RTX (Repeats**-**in**-**Toxin) family of bacterial proteins and is a pore**-**forming toxin. Its expression is regulated by both, environmental and genetic factors, however, the exact expression trigger is unknown. Macrophages are the cells that are most sensitive to LtxA**-**induced cell lysis, which leads to the release of high amounts of IL**-**1β, a strong proinflammatory mediator (136, 137). It was shown, that LtxA induces hypercitrullination in neutrophils, and leukotoxic *A. actinomycetemcomitans* strains were found associated with both, PD and RA. Despite association between exposure to *A. actinomycetemcomitans* and ACPA in RA, the presence of *A. actinomycetemcomitans* cannot be attributed solely to RA (138). In a recent case study reported by Mukherjee et al. (139), a 59**-**year**-**old man with ACPA**-**positive RA was diagnosed with highly leukotoxic strain of *A. actinomycetemcomitans* endocarditis. After treatment with a ceftriaxone, an antibiotic from the beta-lactam group, previously elevated levels of ACPA and CRP normalized, joint symptoms resolved and did not reappear for one year after the treatment.

### C. curtum

There are many other bacteria in the oral microbiota, and these have been suggested as possible factors that contribute to development of RA. Recently, *C. curtum* was identified as a predominant component of the oral microbiota of patients with RA (90). This species is an oral pathogen, however, its translocation can cause several infections, including pelvic abscesses, gynaecologic infections and wounds (140). It is a Gram**-**positive, anaerobic, asaccharolytic rod, which has an ability to produce citrulline along with ornithine and ammonia due to arginine degradation (141). *C. curtum* can be found in the oral and gut microbiota of patients with early RA (87).

### F. nucleatum

*F. nucleatum* is a periodontal bacterium that facilitates and promotes growth of other periodontal pathogens, thus it contributes indirectly to RA development. This early colonizing anaerobic bacterium belonging to the *Bacteroidaceae* family is a dominant species in the periodontium (142). *F. nucleatum* interacts with both, Gram**-**positive and Gram**-**negative bacteria present in dental biofilms (143, 144). An increased number of *F. nucleatum* are found at sites of PD, however, it is not responsible directly for periodontal tissue destruction associated with PD (145). Several studies show that co**-**infection with *F. nucleatum* increases attachment and invasion of human gingival epithelial cells by *P. gingivalis* and *A. actinomycetemcomitans* (146, 147). These phenomena are inhibited by galactose. Another example of a synergistic interaction between *F. nucleatum* and *P. gingivalis* is that co-infection with these two bacterial species increases alveolar bone loss to a greater extent than infection by either bacterium alone (14). However, interactions between bacteria are not always synergistic. Recent study has demonstrated, that collagen**-**induced arthritis in mice inoculated with a mix of *P. gingivalis*, *F. nucleatum* and *A. actinomycetemcomitans* showed less alveolar bone loss compared to mice inoculated with *P. gingivalis* alone. At the same time, *F. nucleatum* and *A. actinomycetemcomitans* alone accelerated the onset and progression of RA (148). Moreover, *F. nucleatum* reacts to host immune responses in PD. Unlike other Gram**-**negative bacteria, *F. nucleatum* is susceptible to host β**-**defensins, particularly β-defensin 3 (hβD**-**3) and LL**-**37 (145). Therefore, *F. nucleatum* is thought to downregulate expression of hβD**-**1 and LL**-**37, nevertheless, hβD**-**2 and hβD**-**3 are upregulated after epithelial exposure to this bacterium (145). Genes upregulated by *F. nucleatum* include those encoding host antimicrobial peptides and proteins, as well as chemokines (IL**-**8, CXCL1, 3, 5 and 10), which attract neutrophils, monocytes, lymphocytes and macrophages, and CSF2 and **-**3, which stimulate neutrophil development (145). Additionally, *F. nucleatum* secretes serine proteases, which enhance its pathogenicity. Like proteases from other periodontal pathogens, such as *P. gingivalis* and *T. denticola*, they are capable of degrading extracellular matrix proteins, such as fibrinogen, fibronectin and collagen type I and IV (145, 149, 150). Moreover, the 65 kDa protease cleaves the α**-**chains of immunoglobulin A, but not immunoglobulin G, which makes it particularly suited to the oral environment. However, it should be noted that, in contrast to the high proteolytic activity of other periodontal bacteria, the activity of this protease is relatively low. To reach the same activity as the phenylalanine**-**specific protease of *T. denticola*, the purified protease from *F. nucleatum* requires nearly ten times longer incubation. This can be explained by the fact, that *F. nucleatum* can be found together with microorganisms showing strong proteolytic activity; therefore, it does not itself require a highly active protease (145).

### F. alocis

*F. alocis* is a very recently discovered Gram**-**positive anaerobic rod, which may play a significant role in development of PD (20). It has several unique characteristics responsible for mediating its pathogenic activity*. F. alocis* is often found in patients with PD, and in contrast to other periodontal pathogens, the species is absent from healthy subjects (151). This suggests, that *F. alocis* plays an important role in development of inflammation and is associated with several oral infections, for example, endodontic infections and in peri**-**implantitis (152, 153). *F. alocis* interacts with other microbial species present in dental biofilms, and these interactions enhance its invasive capacity, which may result in chronic inflammation. It has been shown, that this bacterium accumulates preferentially at sites rich in *F. nucleatum*, but cannot colonize niches occupied by Gram**-**positive streptococci, *e.g.*, *Streptococcus gordonii*. Interactions with *A. actinomycetemcomitans* are strain**-**dependent, but some strains of *A. actinomycetemcomitans* facilitate accumulation of *F. alocis* (154). Moreover, *F. alocis* induces release of host proinflammatory cytokines resulting in apoptosis of gingival epithelial cells and most importantly, it has an ability to adhere to and invade epithelial cells, which is enhanced in the presence of *P. gingivalis*, although the exact mechanism underlying their interaction remains unclear (155, 156). Co**-**culture of *P. gingivalis* and *F. alocis* results in a significant increase in biofilm formation. It is driven by the fact, that *F. alocis* shows very high resistance to oxidative stress. Therefore, its presence increases the survival of *P. gingivalis* under oxidative stress conditions (156). *F. alocis* also exhibits increased activity of non**-**gingipain**-**type proteases (its gingipain**-**type activity is low), that primarily recognize arginine and lysine at a cleavage site. *In silico* studies of the *F*. *alocis* genome revealed the presence of multiple genes involved in degrading arginine to ornithine and citrulline (20). Due to the presence of specific deiminases, *F. alocis* decomposes a C**-**terminal arginine to ornithine and ammonia, which counteracts acidic conditions generated by carbohydrate metabolism in biofilms (157).

### P. intermedia

*P. intermedia* is a Gram**-**negative, obligate anaerobic bacterium associated with PD (158). It is found in both, healthy and inflamed periodontal tissue in PD patients. This species shows high degradative enzyme activity, which also contributes to progression of PD (159). *P. intermedia* expresses nucleases that degrade neutrophil extracellular traps, leading to release of endogenous PADs (160, 161). Although *P. intermedia’s* DNA along with antibodies against this pathogen were detected in synovial fluid from patients with RA, infection did not exacerbate collagen**-**induced arthritis (82, 84, 162). Moreover, a study on Lewis rat model has demonstrated, that *P. intermedia* causes only mild gingivitis; moreover, bone erosion was not observed and rats did not develop systemic inflammation. However, antibodies against *P. intermedia* were detected in rats serum after 1 month of exposure and their concentration increased after 4, and 8 months, respectively (110). A recent study identified a new citrullinated peptide of cytokeratin 13 (CK13**-**1) in GCF against which RA patients mounted an antibody response. It should be noted, that anti**-**cCK13**-**1 antibodies, together with anti**-**cTNC5 (tenascin C) antibodies are associated with the presence of *P. intermedia*.

**Figure 3:**
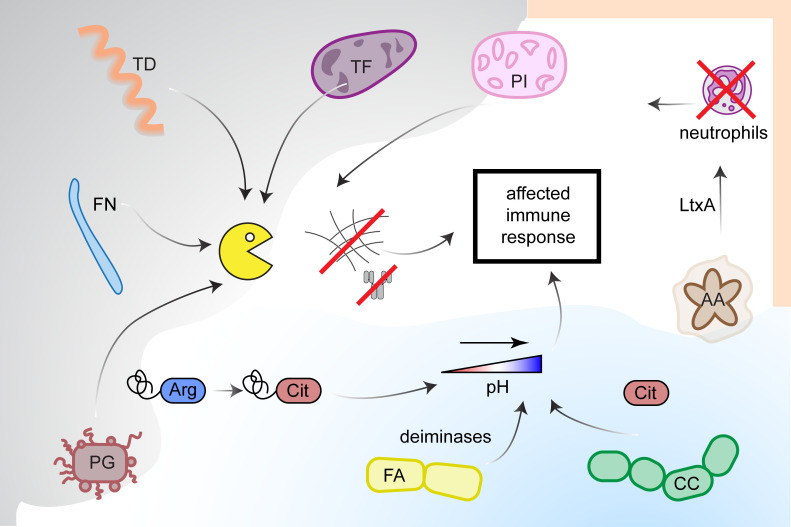
Modulation of immune response by periodontal pathogens. Host immune response can be affected in several ways. Proteases (yellow icon) secreted by *Fusobacterium nucleatum* (FN), *Prevotella intermedia* (PI), *Treponema denticola* (TD), *Tannerella forsythia* (TF) and *Porphyromonas gingivalis* (PG) cleave proteins of extracellular matrix, what leads to tissue degradation. Moreover, PG secretes peptidylarginine deiminase (PPAD), which citrullinates C-terminal arginines of proteins and peptides creating new epitopes recognized by anti-citrullinated proteins antibodies (ACPA). Activity of PPAD together with deiminases from *Filifactor alocis* (FA) and *Cryptobacterium curtum* (CC) contributes to the increase of pH in oral cavity. Leukotoxin A from *Aggregatibacter actinomycetemcomitans* (AA) affects white blood cells (mostly macrophages) inducing their lysis and stimulation of NETs release from neutrophils, which in turn are degradated by nucleases expressed by PI. Degradation of NETs leads to endogenous PADs release.

Brief summary of modulatory effects exerted by periodontal pathogens on host immune response is presented in **Figure 3**.

## Molecular mechanisms linking PD and RA

### Rheumatoid factors

Rheumatoid factors (RFs) are antibodies specific for the Fc region of IgG, and comprise immunoglobulins of any type, however, predominantly belong to the IgM class (163). RFs were first discovered in RA patients over 70 years ago, and since then, together with ACPA, they have been used as diagnostic and predictive markers for RA. Although RFs are associated mainly with RA, they are also present in those with other autoimmune and inflammatory diseases, as well as in healthy individuals (164). Moreover, RFs are detected in the gingiva, subgingival plaques and serum of patients with PD, and in seropositive patients cross**-**react with bacterial epitopes (165–167). Gingipains, an important virulence factor of *P. gingivalis*, are responsible for epitope exposure in the RF**-**Fc region. The Fc regions of IgG molecules contain sequences of lysine and arginine, thus gingipains play a role in IgG3 CH2 and CH3 domains processing and have a key function in RFs production (168). It should be also mentioned, that IgG Fc regions in RA patients harbour significantly fewer galactose residues than those in age**-**matched healthy controls, thus lack of terminal galactose residues may play a role in more severe disease progression (169). *P. melaninogenica* is a saccharolytic bacterium, that metabolises galactose molecules, then it binds to the Fc region of IgG antibodies and metabolises galactose residues (170). The resulting alteration in the composition of sugar moieties affects the activity of antibodies associated with autoimmune diseases.

### Antigens and autoantibodies

Recently, autoantigens involved in RA disease pathology have been well studied and characterized. Interests are focused mainly on citrullinated proteins as true autoantigens in RA. It is assumed, that inflammation and proinflammatory mediators activate PADs in calcium**-**rich environments *via* cleavage and activation of human proteinase**-**activated receptor**-**2 (PAR**-**2), which contributes greatly to accumulation of citrullinated proteins (171, 172). At present, there are four well established citrullinated autoantigens, *i.e.*, collagen type II, fibrinogen, vimentin and α**-**enolase. However, Wegner et al. (39) argue, that identification of all citrullinated proteins is necessary before we can fully explore pathogenic mechanisms. Serum ACPAs are present in 70% of patients with RA (173, 174). They are associated with RA progression and can be detected 10 years before onset of clinical symptoms, and also correlate with progression of PD (175). Based on these findings, there is a hypothesis, that citrullination associated with PD in genetically susceptible subjects can lead to localised oral immune responses, which may result in systemic ACPA responses followed by synovial inflammation and onset of RA (176). However, Konig et al. (177) argue, that the central role of ACPA is to break tolerance during RA development and they propose, that loss of tolerance is a consequence of the presence of antibodies against native proteins, which precede generation of ACPA.

Another post**-**translational modification that can affect protein structure and function is carbamylation. This involves non**-**enzymatic binding of cyanate to the primary amine of lysine, which forms a carbamyl group to generate homocitrulline. Carbamylated proteins induce production of autoantibodies (anti**-**CarP) (178). Neutrophils and their associated myeloperoxidases (MPOs) contribute to carbamylation by promoting conversion of thiocyanate to cyanate (179). Anti**-**CarP antibodies can predict RA development independently of anti**-**CCP2 (citrullinated cyclic peptide 2) antibodies. Their presence can be detected in both, ACPA**-**positive and ACPA**-**negative pre**-**RA patients, and in those with established RA. Detection of anti-CarP in ACPA-negative patients may indicate a more severe disease course (180). Carbamylated proteins have been detected in inflamed gingival tissue, along with elevated levels of MPO, however, there is no significant correlation between PD and anti**-**CarP (181, 182). Moreover, there is no association between anti**-**CarP and RA risk factors, either genetic or environmental, *e.g.*, smoking (183).

Patients with established RA show elevated levels of antibodies against proteins modified with malondialdehyde**-**acetaldehyde adducts (MAAs), which are also associated with ACPA and RFs (184). MAAs result from modification of lysine residues in proteins, which is mediated by highly reactive malondialdehyde and acetaldehyde molecules formed by ROS during lipid peroxidation. ROS are generated mainly under conditions of oxidative stress, however, they can also be released by neutrophils (185). There are indications, that *P. gingivalis* may contribute to production of anti**-**MAA antibodies in mice (186).

### Citrullination

PPAD contributes to RA development by generating citrullinated antigens. Evidence suggests, that human fibrinogen and α**-**enolase (targeted by ACPA in RA) are substrates for PPAD, and that antibodies against citrullinated α**-**enolase from *P. gingivalis* cross**-**react with human anti**-**α**-**enolase antibodies (187). It is suggested, that PPAD can autocitrullinate its own arginine residues, however this was observed only in a single case of PPAD expressed by *E. coli* (188). Anti**-**PPAD antibodies detected in RA are not directed against citrullinated PPAD (189). There is no consensus concerning the role of PPAD and anti**-**PPAD antibodies in RA. According to the study by Quirke et al. (188), anti**-**PPAD antibody levels in patients with RA are higher than those in controls. However, another study showed no correlation between these antibodies and ACPA levels or severity of RA, where the ACPA levels decreased in patients with both, RA and PD (89). The discrepancy between these studies may be due to methodological differences. Recently, another study showed a correlation between anti**-**PPAD IgG and anti**-**CCP IgG in RA patients treated with disease**-**modifying anti**-**rheumatic drugs (DMARDs). Subjects with lower anti**-**PPAD levels showed better responses to DMARDs treatment than those with higher levels (190). These results may promote anti**-**PPAD antibody levels as a tool for predicting responses to therapy. Autoantibody profiles may influence treatment responses. Indeed, patients with a broad baseline of autoantibodies respond better during early stages of treatment, however, they are less likely to achieve initial drug**-**free remission (191). A broad baseline of autoantibodies may be due to more active humoral immune responses to several different antigens and to switching of antibody isotypes. This process can be targeted efficiently by medications (191). The hypothesis of PPAD as a possible link between PD and RA is supported by studies using animal models. Mice infected with a *P. gingivalis* strain expressing PPAD developed collagen**-**induced arthritis more rapidly than controls, and the course of disease was more severe. Moreover, the levels of citrullinated proteins at disease sites were higher (162). Although the link between PD and RA is well established, the particular role of *P. gingivalis* and its PPAD is less clear. PD is a polymicrobial disease, therefore, interaction between *P. gingivalis* and other oral bacteria should be taken into account. For example, *A. actinomycetemcomitans* produce pore**-**forming LtxA, which induces hypercitrullination in neutrophils. Deregulation of neutrophil**-**citrullinating enzymes by LtxA may result in neutrophil trap formation and subsequent release of hypercitrullinated proteins and peptides. Moreover, the presence of LtxA**-**producing *A. actinomycetemcomitans* was confirmed in RA patients, and correlated with ACPA and RF (138). In addition, a recent study postulates, that *A. actinomycetemcomitans* is able to induce autoimmune responses associated with RA in genetically predisposed individuals (139). However, it should be noted, that there is a negative association between *P. gingivalis* and *A. actinomycetemcomitans* in subgingival samples (192). In addition, *P. gingivalis* inhibits growth and attachment of *A. actinomycetemcomitans* within co**-**cultured biofilms (193, 194). Furthermore, the presence of *P. gingivalis*, *P. intermedia* and *P. nigrescens* inhibits activity of LtxA (195).

### Heat shock proteins (HSPs)

HSPs interact reversibly with non**-**folded abnormal proteins and peptides, thereby protecting the cell from proteotoxic stress. They play a role in innate and acquired immune responses and are associated with RA pathogenesis. Bacterial HSPs were found at high levels in serum samples from patients with RA, and the 70 kDa HSP of *P. melanogenica* and *P. intermedia* was found in serum samples from patients with PD (196, 197). Interestingly, antibodies specific for HSP70 and HSP40 of *A. actinomycetemcomitans* are present in the synovium of patients with RA (196). Expression of HSP70 is triggered by stress factors, such as heat, trauma, endotoxins and anti**-**inflammatory drugs, but its expression in synovium is mainly triggered by proinflammatory cytokines, such as TNFα, IL**-**1 and IL**-**6 (198). Therefore, these results may indicate a role of HSPs produced by *A. actinomycetemcomitans*, but not other oral bacteria in patients with RA.

### Immune cells

Neutrophils play an important role in both, PD and RA, as they are responsible for deregulated immune responses that result in tissue damage. Moreover, they contribute to production of autoantibodies. Neutrophils in patients with PD, RA and other inflammatory diseases display an activated phenotype and high levels of NETs (neutrophil extracellular traps) release. Oral bacteria promote neutrophil**-**mediated production of autoantibodies. Several factors are involved in this process, *i.e.*, LtxA from *A*. *actinomycetemcomitans*, degranulation of neutrophils promoted by *F. alocis*, and release of NETs triggered by *P. gingivalis*, *A. actinomycetemcomitans* and *F. nucleatum* (199–201).

The common feature of inflamed tissue in PD and RA is infiltration by high numbers of other immune cell types, including dendrocytes, macrophages and B and T lymphocytes. Joint and blood samples from patients suffering from RA harbour unusual populations of CD4+CD28**-** T cells (202). These cells are similar to NK T cells and have the ability to produce large amounts of IFN γ (interferon γ), which is a marker of activated T cells (known as T_H_1 helper cells) and contain intracellular perforin and granzyme B, providing them with the ability to lyse target cells. Their outgrowth into large clonal populations may be partially attributed to a defect in down-regulating Bcl-2 when deprived of T cell growth factors. In the absence of the CD28 molecule, these unusual CD4 T cells use alternate costimulatory pathways, while the secretion of pro-inflammatory factors stimulates the development of RA (202). T_H_1 cells play a role in promoting autoimmune diseases (203). Moreover, IFN γ increases expression of MHC class II and stimulates production of proinflammatory mediators by macrophages (204). Th17 cells are another T cell subset found at sites of chronic inflammation in PD and in the synovium of RA patients (205). These cells secrete IL**-**17, which plays an important role in RA pathogenesis. In brief, IL**-**17 promotes production of proinflammatory cytokines by macrophages and fibroblasts, facilitates infiltration of joints by immune cells, induces expression of matrix metalloproteinases, and also contributes to bone resorption (206). It has been shown that *P. gingivalis* antigens induce expression of IL**-**17 (207).

Plasma cells and B cells are the most common cell types found in periodontal lesions comprising about 50% and 18% of all immune cells, respectively (208). *P. gingivalis* promotes B cell hyper**-**reactivity by stimulating dendritic cells (209). In addition, B cell survival and maturation into plasma cells is enhanced in gingival epithelium. The presence of B cells in periodontal tissue is crucial for maintaining periodontal health. Antibodies secreted by plasma cells control bacterial growth *via* neutralisation, opsonisation and complement activation. However, hyper**-**reactivity of B cells may be harmful due to their ability to present antigens effectively to T cells, which leads to osteoclastogenesis and increased bone resorption (210).

## Conclusions

We summarized herein epidemiological studies that establish a correlation between PD and RA on multiple levels. While many studies show higher prevalence and severity of PD in patients with RA, others demonstrate that patients with PD are more prone to developing RA. However, since correlation does not imply causation, the precise mechanisms connecting these two diseases remain unclear.

Both, PD and RA share the same risk factors, including HLA**-**DRB1**-**04 as a genetic factor, smoking and infection with EBV and cytomegalovirus. Moreover, both conditions are characterized by chronic inflammation, a crucial role played by B cells and plasma cells, and tissue destruction evidenced by alveolar bone resorption and joint erosion. These common features may suggest a similar underlying mechanisms of both diseases.

Furthermore, we discussed in this review the associations among bacteria responsible for the onset, development and progression of PD and RA. We focused mainly on oral pathogens, which are designated as “red complex” bacteria, and are established the aetiological agents of PD. We also attempted to cover the role of other, less commonly described bacterial species present in the oral microbiome of patients with PD and/or RA. We analyzed the main virulence factors of these microorganisms, their mechanisms of action and their effects on host immune responses. We strived to provide examples of cross**-**talk between bacterial pathogens and to indicate potential overlap of pathogenic mechanisms that may lead to synergistic effects. In general, the evidence presented herein supports a dominant paradigm involving a microbiome shift that results in PD and, possibly, RA. Hopefully, future in depth investigations of the oral microbiome and the molecular mechanisms utilized by oral bacteria will pave the way for novel treatments and diagnostic tools for PD and RA, so far, two incurable diseases.

## Author contributions

AK, conceptualization, data acquisition and analysis, and writing-original draft preparation; KS and AD, literature search, data analysis and visualization, writing-editing; GPB, data acquisition and analysis, writing-reviewing, and editing; KŁ-B: design, data analysis, writing-reviewing and editing; JP, conceptualization and design, writing-editing, and supervision; KG, conceptualization and design, writing-original draft preparation, and supervision; All authors contributed to the article and approved the submitted version.

## Acknowledgments

We thank the National Science Centre of Poland (UMO-2018/29/B/NZ2/01930 to KG, UMO-2018/30/A/NZ5/00650 to JP) and NIH/NIDCR (DE 022597 to JP) for providing funding to many of the experiments reviewed in this publication.

## Conflict of interest

The authors declare that the research was conducted in the absence of any commercial or financial relationships that could be construed as a potential conflict of interest.

## Publisher’s note

All claims expressed in this article are solely those of the authors and do not necessarily represent those of their affiliated organizations, or those of the publisher, the editors and the reviewers. Any product that may be evaluated in this article, or claim that may be made by its manufacturer, is not guaranteed or endorsed by the publisher.
